# Supplementary material for: Exosomal Long Non-Coding Ribonucleic Acid Ribonuclease Component of Mitochondrial Ribonucleic Acid Processing Endoribonuclease Is Defined as a Potential Non-Invasive Diagnostic Biomarker for Bladder Cancer and Facilitates Tumorigenesis via the miR-206/G6PD Axis
Source: Cancers (Basel). 2023 Nov 6;15(21):5305. doi: 10.3390/cancers15215305 (PMC10649581; doi:10.3390/cancers15215305)
Supplement: Supplementary file 1 [file cancers-15-05305-s001.zip › cancers-15-05305-s001/SUPPLEMENTARY FIGURES AND MATERIALS.pdf]

SUPPLEMENTARY FIGURES AND FIGURE LEGENDS

FIGURE LEGENDS

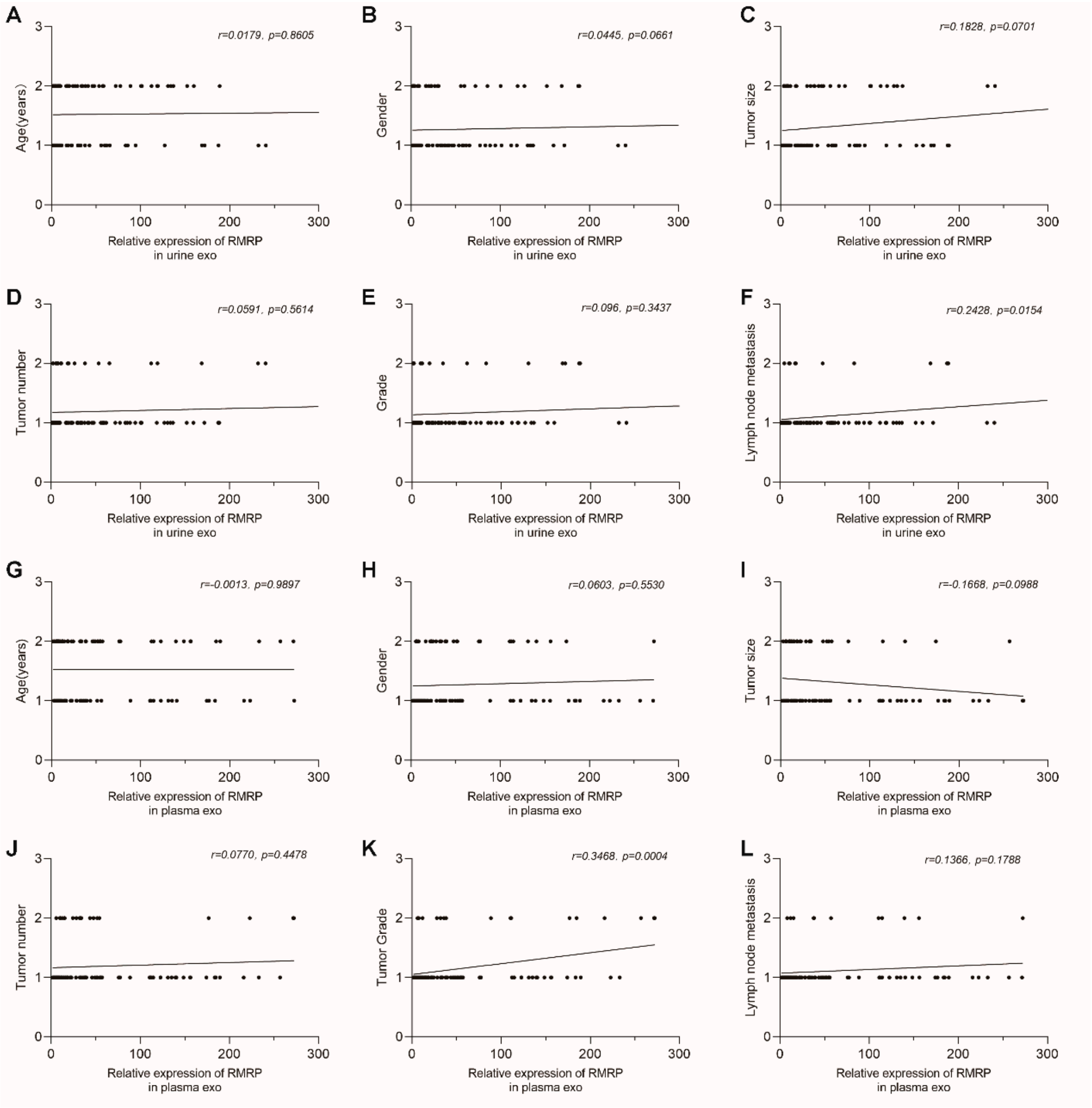

Supplementary Figure S1

No significant correlation between RMRP expression (A-F) in urine or (G-L) plasma exosomes and most clinical characteristics of BLCA patients. exo: exosome. The correlation of RMRP expression with (A and G) age, (B and H) gender, (C and I) tumor size, (D and J) tumor number, (E and K) tumor grade and (F and L) lymph node metastasis in BLCA patients.

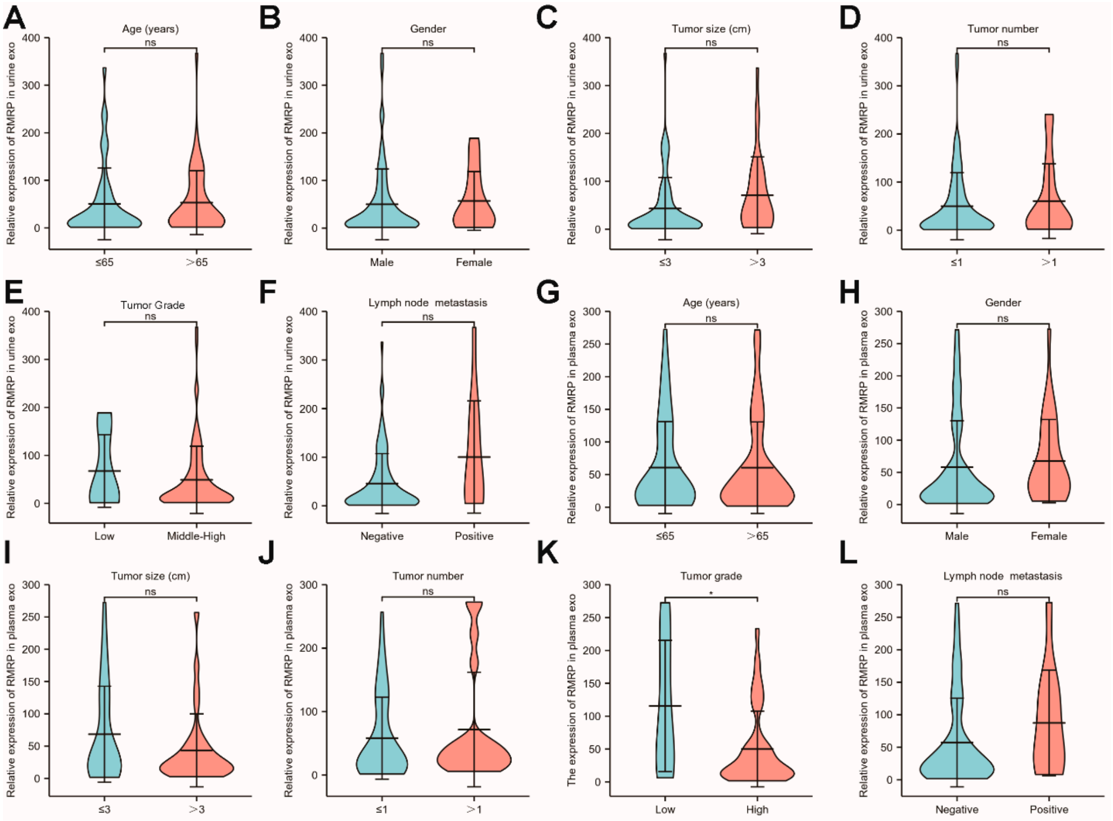

Supplementary Figure S2

Associations between RMRP expression in (A-F) urine exosomes or (G-L) plasma exosomes and paired BLCA tissues. The RMRP expression in different groups from (A and G) age, (B and H) gender, (C and I) tumor size, (D and J) tumor number, (E and K) tumor grade and (F and L) lymph node metastasis in BLCA patients. ns: $p > 0.05$, $^{*}p < 0.05$.

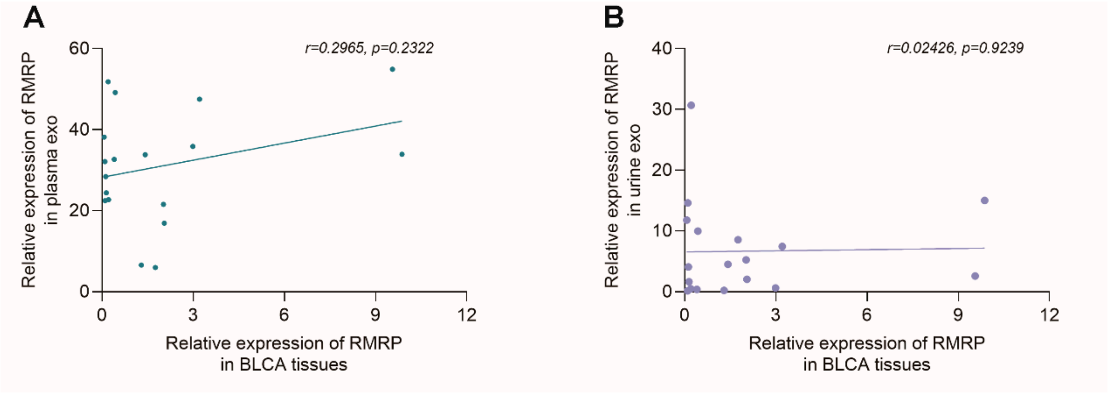

Supplementary Figure S3

The correlation of RMRP relative expression in (A) plasma-exosome or (B)urine-exosome and tumor tissues of BLCA patients. exo: exosome.

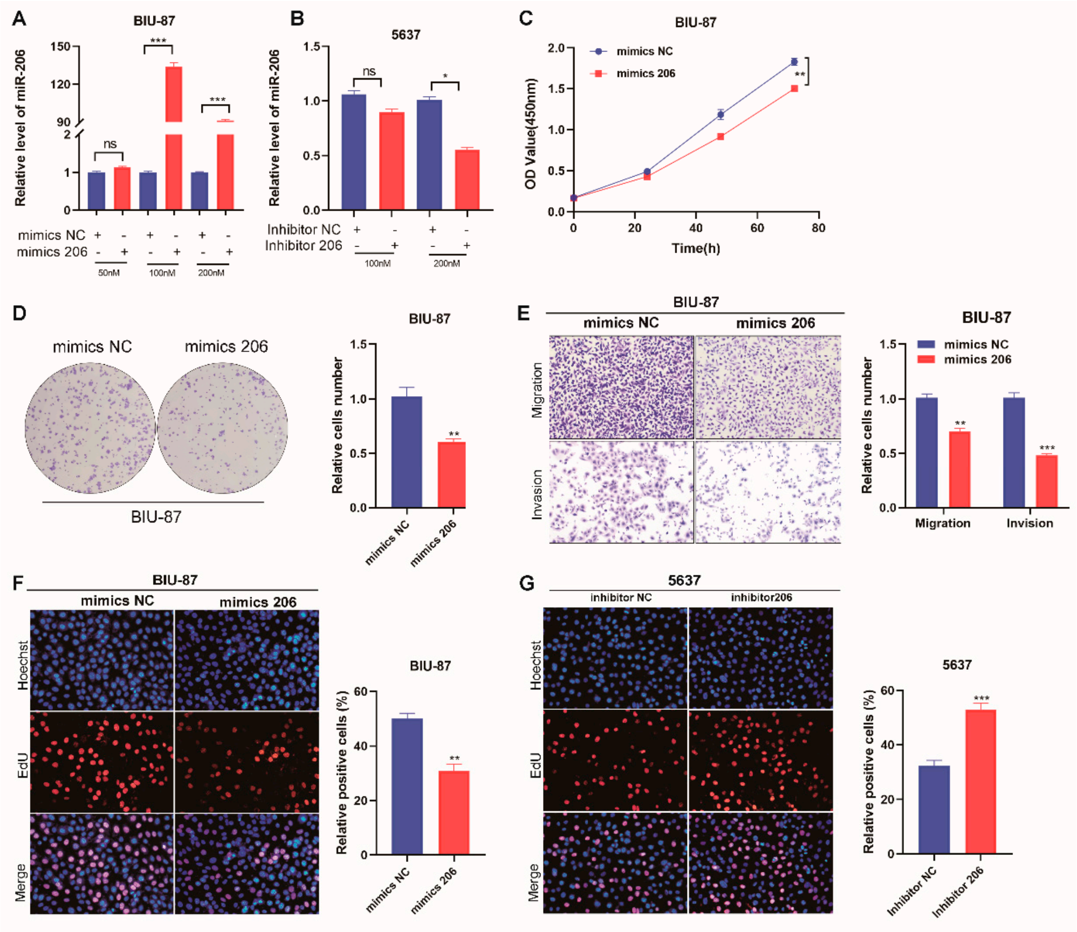

Supplementary Figure S4

(A)The relative expression of miR-206 in BIU-87 cells treated with mimics NC or mimics 206 at different concentrations (50,100 and 200nM). (B) The relative expression of miR-206 in 5637 cells treated with mimics NC or mimics 206 at different concentrations (100 and 200nM). (C) Cell proliferation of BIU-87 cells treated with mimics NC or mimics 206 was determined by CCK-8 at 0, 24, 48, and 72h. (D) Colony formation assays of BIU-87 cells treated with mimics NC or mimics 206were detected at day 14. (E) Cell migration and invasion assays using Transwell in BIU-87 cells treated with mimics NC or mimics 206. (F and G) EdU assays were used to detect the proliferation rate of BIU-87 cells treated with (F) mimics NC or mimics 206 and (G)inhibitor NC or inhibitor 206. Columns are the average of three independent experiments. All data are presented as the mean ± SEM of triplicate experiments. $^{**}p < 0.01$, $^{***}p < 0.001$.)

SUPPLEMENTARY MATERIALS AND METHODS

Cell culture

BIU-87 and 5637 cultured in RPMI-1640 medium (Invitrogen, USA) with 10% FBS (Gibco, USA) and maintained in an atmosphere containing 5% CO_2. To determine the effect of miR-206 on tumor progression in BLCA cells, BIU-87 or 5637 cells were treated with mimics NC, mimics 206 or inhibitor NC and inhibitor 206.

CCK8 assay

BIU-87 cells were seeded at a density of 2000 cells per well in a 96-well plate (Corning, NY, USA) treated with mimics NC or mimics 206. At the specified time points (24 h, 48 h, 72 h), 10 μL of CCK-8 reagent (Dojindo Crop, Japan) was added to each well containing the culture medium. Then cells were incubated at 37 °C for 2 hours. OD value was measured at 450 nm using a microplate reader (Synergy H4 Hybrid Reader, BioTek, USA). These experiments were repeated three times.

Colony formation assay

BIU-87 cells treated with mimics NC or mimics 206 were seeded in six-well plates at a density of 2000 cells per well and cultured for 14 days. Subsequently, the cells were rinsed with PBS three times, fixed with methanol, and stained with 0.5% crystal violet (Kaigen, China) for 15 minutes. Finally, the colonies were counted and recorded. These experiments were replicated three times.

Transwell assays

BIU-87 cells were pre-treated with mimics NC or mimics 206 for 24h. Then cells were suspended in serum-free medium, and 1×10^5 cells were seeded into the upper chambers of Transwell inserts (Corning Glass Works, Corning, NY, United States). The lower chamber was filled with medium containing 10% FBS, serving as a chemoattractant for cell migration assays. After incubation for 24-48 hours, the Transwell chambers were fixed with methanol and stained with 0.5% crystal violet (Kaigen, China) for 15 minutes. Following three washes with PBS, migrated cells were photographed and counted in

five different fields. These experiments were repeated three times. For the cell invasion assay, the procedure was similar to the above, except that the Transwell inserts were pre-coated with a matrigel mixture before cell seeding.

EdU incorporation assay

BIU-87 cells treated with mimics NC or mimics 206 were seeded into 24-well plates at a density of 5×10^4 cells/well, assessed the proliferation of cells by EdU incorporation assay kit (RiboBio, China), following the manufacturer's protocol. The images were acquired using the Zeiss LSM880 NLO confocal microscope (Leica, Germany).

Statistical analysis

Statistical analysis was performed using SPSS (IBM SPSS Statistics, version 25.0, SPSS IBM, NY, USA). BLCA patients were divided into low- (n = 49) and high-expression (n = 50) groups, using the median value of plasma or urine exosome-RMRP expression as the cut-off value. *Pearson's* χ^2 *test* was performed to explore associations between RMRP expression levels in urine and plasma exosomes and BLCA patient clinicopathological characteristics. Using violin plots, the relationships between RMRP expression levels in urine and plasma exosomes and clinical features were analyzed by *t-tests*. All statistical analyses were done using GraphPad Prism software (GraphPad Prism version 8.3.1 for Windows; GraphPad Software, www. graphpad. com). All data are presented as the mean ± SEM. $*p < 0.05$, $**p < 0.01$, $***p < 0.001$ were considered statistically significant.
