# Supplementary material for: Exosomal Long Non-Coding Ribonucleic Acid Ribonuclease Component of Mitochondrial Ribonucleic Acid Processing Endoribonuclease Is Defined as a Potential Non-Invasive Diagnostic Biomarker for Bladder Cancer and Facilitates Tumorigenesis via the miR-206/G6PD Axis
Source: Cancers (Basel). 2023 Nov 6;15(21):5305. doi: 10.3390/cancers15215305 (PMC10649581; doi:10.3390/cancers15215305)
Supplement: Supplementary file 1 [file cancers-15-05305-s001.zip › cancers-15-05305-s001/SUPPLEMENTARY TABLES1.pdf]

SUPPLEMENTARY TABLES

Supplementary Table S1. Clinical information of the patients with BLCA.

Number	Gender	Age (years)	Tumor size (≤3cm(1), ＞3cm(2))	Tumor number	Grade	Lymph node metastasis	Tumor stage (T)	urine cytology	NMP-22	NMP-22 （ELISA) ng/ml	BTA (ELISA) ng/ml
1	Male	74	1	1	High	Negative	Ta-T1	(+)	(-)	1.86	7.27078
2	Male	63	2	1	High	Negative	Ta-T1	(+)	(-)	1.848	9.64534
3	Male	74	1	1	High	Positive	Ta-T1	(+)	(-)	1.92	7.68982
4	Male	47	1	1	High	Negative	Ta-T1	(+)	(-)	1.776	2.31214
5	Female	45	1	1	High	Negative	Ta-T1	(-)	(+)	1.812	4.66924
6	Male	67	1	1	High	Negative	Ta-T1	(-)	(-)	1.908	3.88354
7	Male	71	1	1	High	Negative	Ta-T1	(-)	(-)	1.824	5.50732
8	Male	84	2	1	High	Negative	Ta-T1	(-)	(+)	1.8	3.35974
9	Male	50	2	1	High	Negative	Ta-T1	(+)	(-)	1.656	3.65656
10	Male	71	1	1	High	Negative	Ta-T1	(-)	(-)	1.692	3.1153
11	Male	65	2	1	High	Negative	Ta-T1	(+)	(+)	1.752	1.1947
12	Male	79	1	1	High	Negative	Ta-T1	(+)	(-)	1.668	4.93114
13	Male	56	1	1	High	Negative	Ta-T1	(-)	(+)	1.884	6.55492
14	Male	59	1	1	Low	Negative	Ta-T1	(-)	(+)	1.8	9.45328
15	Male	42	1	1	High	Negative	Ta-T1	(-)	(-)	1.884	2.38198
16	Male	52	1	1	High	Negative	Ta-T1	(+)	(-)	1.74	3.69148
17	Female	52	1	1	High	Negative	Ta-T1	(-)	(-)	1.764	3.86608
18	Male	71	2	1	High	Negative	Ta-T1	(-)	(+)	1.572	6.11842
19	Male	70	1	1	Low	Negative	Ta-T1	(-)	(+)	1.668	4.23274
20	Male	72	1	1	High	Negative	Ta-T1	(-)	(-)	1.824	4.11052

21	Female	63	1	1	Low	Negative	Ta-T1	(+)	(+)	2.004	8.91202
22	Female	46	1	1	High	Negative	Ta-T1	(+)	(-)	1.728	4.79146
23	Male	63	2	Multiple	High	Negative	T2-T4	(-)	(-)	1.656	2.2423
24	Male	76	1	1	High	Negative	Ta-T1	(+)	(-)	1.752	7.84696
25	Female	56	2	1	High	Negative	Ta-T1	(+)	(-)	1.32	7.18348
26	Male	67	2	1	High	Negative	Ta-T1	(+)	(+)	1.86	8.66758
27	Male	81	2	1	High	Negative	Ta-T1	(+)	(+)	1.836	6.6073
28	Male	76	1	1	High	Negative	Ta-T1	(-)	(+)	1.872	2.73118
29	Male	74	1	1	High	Negative	T2-T4	(-)	(+)	1.596	4.52956
30	Male	65	1	1	Low	Negative	T2-T4	(+)	(-)	1.752	9.26122
31	Male	33	1	Multiple	High	Negative	T2-T4	(-)	(+)	1.812	3.60418
32	Male	48	1	Multiple	High	Negative	Ta-T1	(-)	(-)	1.824	2.83594
33	Female	75	1	1	High	Negative	Ta-T1	(+)	(+)	1.86	11.4088
34	Male	62	1	1	High	Negative	Ta-T1	(-)	(+)	1.764	4.63432
35	Male	67	1	1	High	Positive	T2-T4	(-)	(+)	1.596	0.95026
36	Male	63	1	2	Low	Negative	Ta-T1	(-)	(-)	1.872	7.89934
37	Male	69	2	Multiple	High	Negative	Ta-T1	(+)	(-)	1.896	6.15334
38	Male	57	1	1	High	Negative	Ta-T1	(+)	(-)	1.86	9.52312
39	Female	49	1	1	High	Negative	Ta-T1	(-)	(-)	1.776	3.09784
40	Female	54	1	1	Low	Positive	T2-T4	(-)	(-)	1.848	8.47552
41	Male	67	2	1	Low	Negative	T2-T4	(-)	(-)	1.632	5.68192
42	Male	88	1	Multiple	Low	Negative	T2-T4	(-)	(+)	1.692	0.21694
43	Male	81	1	1	High	Negative	Ta-T1	(-)	(-)	1.644	16.62934
44	Female	58	2	1	High	Negative	Ta-T1	(-)	(-)	1.692	7.93426
45	Female	71	2	1	High	Negative	Ta-T1	(-)	(+)	1.836	7.46284

46	Female	49	1	1	High	Negative	Ta-T1	(+)	(-)	1.344	7.2184
47	Female	58	2	1	High	Negative	T2-T4	(+)	(+)	1.872	11.16436
48	Male	87	1	1	High	Negative	T2-T4	(+)	(+)	2.016	6.65968
49	Male	73	1	1	High	Negative	Ta-T1	(+)	(-)	3.204	11.79292
50	Male	69	1	1	High	Negative	Ta-T1	(-)	(-)	1.764	9.8374
51	Female	55	2	1	High	Positive	Ta-T1	(+)	(-)	1.764	4.45972
52	Female	55	2	1	High	Negative	T2-T4	(-)	(-)	1.764	6.81682
53	Female	71	1	1	High	Negative	Ta-T1	(+)	(-)	1.884	6.03112
54	Female	58	1	1	High	Negative	Ta-T1	(-)	(+)	1.848	7.6549
55	Male	52	1	1	High	Positive	Ta-T1	(+)	(-)	1.788	5.50732
56	Female	59	1	Multiple	Low	Positive	T2-T4	(-)	(-)	1.956	5.80414
57	Male	68	2	1	High	Positive	Ta-T1	(+)	(+)	1.908	5.26288
58	Male	72	1	1	Low	Negative	Ta-T1	(+)	(-)	1.74	3.34228
59	Female	68	1	1	High	Negative	Ta-T1	(-)	(-)	1.668	8.66758
60	Male	55	1	2	High	Negative	Ta-T1	(-)	(-)	1.764	10.29136
61	Male	74	1	1	High	Negative	Ta-T1	(+)	(-)	1.92	13.18972
62	Female	53	1	1	High	Negative	Ta-T1	(+)	(+)	1.776	15.14524
63	Male	70	1	Multiple	High	Negative	T2-T4	(-)	(-)	1.752	8.37076
64	Male	71	1	1	High	Negative	Ta-T1	(+)	(+)	1.14	4.6867
65	Male	58	1	1	Low	Negative	Ta-T1	(-)	(+)	1.848	11.63578
66	Male	70	1	1	High	Negative	Ta-T1	(+)	(+)	1.848	9.88978
67	Male	66	1	1	High	Negative	Ta-T1	(+)	(-)	1.524	9.47074
68	Female	58	1	1	High	Negative	T2-T4	(-)	(-)	1.86	3.04546
69	Male	63	1	1	Low	Positive	T2-T4	(-)	(-)	1.848	8.42314
70	Male	72	2	2	High	Negative	T2-T4	(-)	(+)	1.74	5.62954

71	Male	68	2	2	High	Negative	Ta-T1	(-)	(-)	1.056	0.16456
72	Male	42	2	1	High	Negative	T2-T4	(+)	(-)	1.908	7.58506
73	Female	67	1	1	High	Negative	Ta-T1	(+)	(-)	1.416	9.06916
74	Male	46	1	1	High	Negative	Ta-T1	(-)	(+)	1.272	7.00888
75	Male	71	1	1	High	Negative	Ta-T1	(+)	(+)	1.812	3.13276
76	Male	54	1	1	High	Negative	Ta-T1	(+)	(+)	1.596	4.93114
77	Male	55	2	Multiple	High	Positive	T2-T4	(+)	(+)	1.56	9.6628
78	Male	67	2	1	High	Positive	T2-T4	(-)	(+)	1.716	9.2263
79	Male	68	1	1	High	Negative	Ta-T1	(+)	(-)	1.644	6.4327
80	Male	55	1	1	High	Negative	Ta-T1	(-)	(-)	1.8	0.96772
81	Male	54	1	1	Low	Negative	Ta-T1	(+)	(+)	3.024	8.38822
82	Male	73	1	1	High	Negative	Ta-T1	(+)	(-)	1.752	9.87232
83	Male	63	2	1	High	Negative	T2-T4	(-)	(-)	1.596	7.81204
84	Female	56	1	1	High	Negative	Ta-T1	(+)	(+)	1.788	3.93592
85	Male	69	2	1	High	Negative	Ta-T1	(-)	(+)	1.668	5.7343
86	Female	75	2	1	High	Negative	Ta-T1	(-)	(+)	1.824	10.46596
87	Male	67	1	1	High	Negative	T2-T4	(-)	(+)	2.7	10.64056
88	Female	91	1	2	High	Negative	Ta-T1	(-)	(+)	1.812	5.26288
89	Male	55	2	2	High	Negative	T2-T4	(-)	(-)	1.836	7.61998
90	Female	73	2	2	High	Negative	Ta-T1	(-)	(-)	1.968	8.91202
91	Female	83	2	2	High	Negative	T2-T4	(-)	(-)	2.04	8.12632
92	Male	64	2	2	High	Negative	Ta-T1	(-)	(-)	2.136	9.7501
93	Male	71	1	1	High	Negative	Ta-T1	(-)	(-)	1.764	7.60252
94	Male	64	1	1	High	Negative	T2-T4	(+)	(+)	1.98	7.89934
95	Male	85	2	1	High	Negative	T2-T4	(-)	(+)	2.16	7.35808

96	Male	59	1	1	Low	Negative	Ta-T1	(-)	(-)	1.92	5.43748
97	Female	92	1	1	Low	Positive	T2-T4	(-)	(+)	2.28	10.76278
98	Male	71	1	2	Low	Negative	Ta-T1	(+)	(-)	2.784	12.38656
99	Male	75	2	2	High	Negative	Ta-T1	(-)	(-)	2.652	15.28492

Supplementary Table S2. Relevant qPCR primer sequences.

Gene Name	Forward (5′ to 3′)	Reverse (5′ to 3′)
RMRP	TGCTGAAGGCCTGTATCCT	TGAGAATGAGCCCCGTGT
GAPDH	ACCCACTCCTCCACCTTTGAC	TGTTGCTGTAGCCAAATTCGTT
18S RNA	CGTTCTTAGTTGGTGGAGCG	CGCTGAGCCAGTCAGTGTAG

Supplementary Table S3. The expression of RMRP and GAPDH in tumor tissues of BLCA patients.

Number	Ct Value			
	Adjacent (30)		BLCA(n=40)	
	GAPDH	RMRP	GAPDH	RMRP
1	23.52	20.03	22.32	20.53
2	23.24666667	21.54666667	22.52	20.89666667
3	22.53666667	24.23	23.85666667	19.57333333
4	18.53666667	18.34	17.62666667	15.36333333
5	19.81	15.08333333	29.58	25.55
6	32.56666667	30.09333333	25.99333333	20.79666667
7	24.63	22.03	29.63333333	25.05666667
8	22.42666667	19.27666667	18.43666667	17.15333333
9	30.16666667	28.30666667	21.72333333	16.22666667
10	33.25666667	32.59666667	29.95	29.63333333
11	32.41333333	28.06	18.18666667	14.87
12	29.85666667	28.10666667	29.09666667	25.45
13	24.49666667	24.53666667	29.58666667	29.16666667
14	23.03333333	22.33666667	20.94666667	17.95
15	26.25	21.55333333	19.62333333	14
16	24.68666667	19.99333333	19.68	15.47666667
17	18.28666667	14.76666667	26.48666667	20.79
18	25.71	23.14	23.50333333	24.80666667
19	27.42	22.59	23.12333333	17.23
20	25.76	23.89666667	29.47	26.26666667

21	32.77666667	31.17333333	33.76	28.44333333
22	21.81666667	21.02	21.35333333	22.61
23	33.34	32.52666667	27.62666667	22.12333333
24	21.42333333	16.58666667	26.36666667	25.4
25	25.37666667	24.14	21.43666667	16.4
26	24.74333333	20.93333333	25.94	24.44666667
27	21.65	16.68	18.66333333	14.47
28	18.60333333	16.92666667	20.66666667	16.98
29	26.59	28.16	27.19666667	23.13
30	18.64333333	15.43	21.52333333	18.42666667
31			18.37	14.38
32			17.23	17.36
33			14.74333333	13.76
34			19.07333333	13.71666667
35			17.87666667	16.69
36			20.87	14.94
37			21.21	16.4
38			18.59333333	16.92666667
39			13.33	12.60666667
40			18.66	15.23666667

Supplementary Table S4. The expression of RMRP and 18s RNA in urine- and plasma- exosomes

Ct Value in urine and plasma exosomes												
Number	Urine						Plasma					
	Normal (n=84)		Benign lesion (n=50)		BLCA (n=99)		Normal (n=84)		Benign lesion (n=50)		BLCA (n=99)	
	18S	RMRP	18S	RMRP	18S	RMRP	18S	RMRP	18S	RMRP	18S	RMRP
1	14.375	29.49	16.72	30.3	13.11	28.87	12.43	25.555	15.89	28.56	13.97	26.31
2	17.395	30.86	9.16	22.77	16.78	31.55	9.3	22.235	14.78	28.39	14.35	28.88
3	16.875	27.5	14.605	25.96	15.95	28.61	10.92	23.935	12.98	25.89	15.18	28.17
4	12.37	26.48	9.605	23.89	16.82	34.14	12.88	25.04	16.93	29.39	14.82	27.92
5	20.15	32.39	11.115	25.18	13.94	30.78	10.125	23.01	15.98	29.47	14.93	27.86
6	11.77	23.04	12.14	24.66	16.68	30.82	11.2	21.78	14.89	27.39	15.28	27.1
7	12.605	28.01	12.7	24.55	15.72	32.13	12.11	25.02	12.92	28.87	15.02	28
8	13.49	25.49	9.92	21.42	15.45	26.38	13.07	25.155	15.05	28.25	13.92	25.71
9	9.85	23.03	12.005	24.6	17.005	27.59	13.63	27.54	16.45	28.95	14.44	27.11
10	12.545	25.66	11.595	25.8	15.82	31.1	11.265	23.335	14.07	29.4	14.28	28.61
11	11.435	23.99	9.79	22.1	12.32	25.21	12.44	25.81	15.09	29.3	15.09	27.89
12	11.595	24.74	10.405	22.74	15.8	29.68	10.88	23.7	14.87	27.59	15.61	27.76
13	12.225	23.75	11.6	23.24	12.85	28.17	12.015	25.61	16.97	29.91	13.98	28.11
14	11.88	25.16	13.12	24.71	14.22	29.02	11.27	25.13	17.3	26.61	14.23	27.31
15	10.865	21.28	12.02	24.36	15.85	34.62	10.41	23.795	15.7	28.09	16.19	28.16
16	9.685	20.49	9.03	23.39	17.42	32.14	14.815	28.21	18.83	31.63	13.69	27.04
17	9.845	25.43	12.85	22.66	15.06	29.75	10.55	22.295	17.01	31.02	15.1	27.08
18	12.95	26.45	11.265	25.57	8.545	17.88	10.25	24.84	12.3	27.42	14.14	26.59
19	11.875	26.72	10.195	24.26	16.74	31.75	12.28	25.125	15.91	27.29	14.46	27.8

20	12.95	30.31	13.495	25.56	18.345	25.5	12.805	25.25	13.9	28.37	14.42	27.31
21	16.19735	30.91175	11.39	23.72	15.22	30.8	12.38	25.99	14.78	25.55	15.81	27.01
22	11.81	26.41	12.825	24.45	16.05	32.68	13.095	25.26	12.18	27.46	14.47	27.71
23	11.55	25.81	9.88	20.31	16.4	31.54	13.085	25.695	12.12	25.28	14.64	27.25
24	11.385	24.41	11.61	22.51	15.84	30.81	12.335	25.135	16.87	30.46	14.91	27.35
25	12.375	22.54	12.85	24.82	14.99	28.14	10.57	20.965	12.09	27.17	15.44	26.93
26	12.6	25.42	12.785	25.66	10.18	20.66	11.565	25.63	14.76	25.29	15.33	27.1
27	12.89	26.04	9.95	24.48	16.31	30.04	14.88	28	13.98	27.39	14.67	26.5
28	10.28	23.49	10.95	20.35	13.04	24.77	10.99	23.945	14.34	26.46	15.105	24.165
29	11.735	24.82	13.955	27.53	12.725	25.66	11.575	24.745	16.14	28.55	14.965	23.75
30	12.99	27	13.705	23.53	10.175	24.75	11.33	24.195	11.54	25.005	15.61	23.855
31	11.17	28.84	17.89	28.4	15.49	30.48	18.255	30.2	13.045	25.655	12.36	20.56
32	14.385	24.78	14.88	28.58	14.71	26.49	18.83	32.505	22.05	34.53	10.52	22.64
33	11.61	24.64	11.76	24.85	12.48	26.45	11.985	27.925	15.815	29.21	15.02	24.21
34	11.07	26.38	11.81	23.72	14.06	27.46	12.265	28.92	18.43	31.475	14.57	24.765
35	15.835	28.02	14.765	27.35	17.03	28.51	12.93	32.84	16.54	28.91	14.15	22.865
36	11.685	26.26	12.91	26.86	13.97	26.46	12.97	29.04	17.055	29.52	14.035	22.57
37	13.86	26.91	12.815	27.31	18.24	28.99	15.04	30.935	14.344	27.344	14.11	24.355
38	12.895	27.38	12.71	25.95	17.91	27.35	13.12	29.21	13.69	26.19	14.23	22.71
39	12.135	26.82	11.745	24.96	17.45	27.55	13.4	28.33	17.46	32.76	11.18	21.5
40	11.935	25.57	10.845	24.59	18.25	26.7	13.13	29.915	12.36	29.76	12.85	22.06
41	11.705	24.33	12.01	24.83	19.24	32.21	12.52	31.155	17.3	32.6	13.55	21.545
42	10.06	24.43	15.975	26.64	14.74	26.44	13.085	30.085	11.9	24.8	15	22.915
43	9.85	23.84	9.96	21.09	13.96	25.78	14.18	28.24	13.8	29.1	13.005	28.24
44	9.74	21.5	11.635	26.93	11.19	25.39	14.83	28.92	17.4	31.7	13.17	26.77

45	13.555	24.78	10.815	23.38	13.14	22.49	12.815	30.135	16.8	32.1	12	21.755
46	5.57	20.03	13.415	26.56	15.7	27.18	13.415	31.045	15.4	30.32	15.175	25.87
47	9.175	21.73	10.315	22.58	18.56	28.76	14.565	28.66	13.9	32.3	14.82	25.81
48	9.865	23.45	12.855	27.26	19.71	28.82	13.425	28.675	14.5	29.8	13.425	21.86
49	12	24.51	10.755	24.02	15.93	30.73	13.365	30.73	14.98	28.07	14.205	22.34
50	10.175	22.34	12.45	26.36	14.22	26.91	13.2	29.3	13.75	28.04	13.905	24.63
51	10.68	23.08			13.85	25.77	13.17	30.81			13.07	22.23
52	11.83	23.93			19.5	28.51	12.125	28.785			17.46	26.015
53	13.78	24.33			16.78	28.75	12.56	28.495			16.16	25.885
54	10.88	23.28			15.84	27.38	13.01	27.63			12.92	21.885
55	11.53	24.47			14.86	28.55	14.36	27.54			16.03	26.79
56	10.04	24.85			19.03	27.63	15.09	29.28			13.67	21.58
57	10.755	24.02			8.145	20	15.78	29.81			13.18	22.055
58	10.89	24.25			17.49	27.37	15.87	29.47			14.96	23.43
59	9.605	23.46			17.06	25.81	16.94	28.67			13.98	22.69
60	9.845	23.49			13.03	26.61	14.78	28.37			15.16	25.715
61	11.01	24.81			17.26	26.79	17.76	31.48			14.4	27.54
62	10.98	24.86			17.93	27.51	14.87	28.59			13.48	25.06
63	10.245	24.84			18.47	28.73	16.7	29.44			13.87	27.335
64	12.7	26.65			17.79	31.91	15.89	28.26			14.36	24.84
65	9.86	23.74			17.61	27.66	15.89	28.57			15.87	25.4
66	10.015	24.05			13.02	26.14	14.28	27.39			14.63	24.855
67	10.255	25.49			12.005	27.01	13.48	29.48			13.28	23.58
68	10.2	24.78			17.22	27.33	14.87	28.04			15.77	24.635
69	10.785	24.4			10.93	23.55	14.05	27.4			14.835	27.26

70	11.02	24.16			18.22	27.41	15.87	29.47			12.51	25.19
71	8.905	22.3			16.27	31.32	14.43	27.81			14.33	26.69
72	15.96	27.56			16.75	27.86	13.78	27.54			14.615	26.785
73	11.57	25.61			16.96	28.64	13.37	27.24			14.94	28.355
74	13.705	25.81			10.595	23.31	14.67	29.49			14.965	29.165
75	13.515	25.98			11.625	24.99	16.8	29.77			14.035	27.775
76	10.845	23.97			14.03	27.44	16.79	28.99			13.55	26.565
77	10.97	23.74			15.77	31.33	16.23	31.11			13.17	25.245
78	11.5	25.16			16.32	26.74	16.97	29.58			16.16	26.32
79	10.65	23.7			13.62	29.76	13.57	28.26			13.97	23.13
80	15.51	28.97			15.73	29.93	15.87	28.7			15.9	24.96
81	11.75	25.72			16.43	31.78	14.67	27.64			16.69	25.89
82	11.79	24.02			14.32	20.45	16.87	29.52			12.27	23.78
83	9.865	24.01			16.415	26.02	16.2	30.68			12.32	23.89
84	11.975	25.76			21.35	28.41	14.05	28.14			13.955	25.45
85					13.72	27.82					13.95	27.235
86					16.07	25.9					12.415	23.335
87					14.35	21.03					14.165	24.545
88					13.65	24.93					16.925	28.315
89					14.35	22.44					13.925	24.84
90					14.65	27.69					14.11	24.415
91					15.4	24.5					10.375	20.805
92					14.82	24.79					13.69	24.86
93					15.74	26.38					16.375	26.595
94					12.83	23.8					15.82	24.74

95					10.335	19.24					14.73	28.15
96					17.49	30.22					11.76	22.595
97					12.73	21.17					13.4	24.145
98					16.925	31.75					13.9	24.895
99					11.98	25.32					14.165	25.135
