# Supplementary figures and images for: Exosomal Long Non-Coding Ribonucleic Acid Ribonuclease Component of Mitochondrial Ribonucleic Acid Processing Endoribonuclease Is Defined as a Potential Non-Invasive Diagnostic Biomarker for Bladder Cancer and Facilitates Tumorigenesis via the miR-206/G6PD Axis
Source: Cancers (Basel). 2023 Nov 6;15(21):5305. doi: 10.3390/cancers15215305 (PMC10649581; doi:10.3390/cancers15215305)

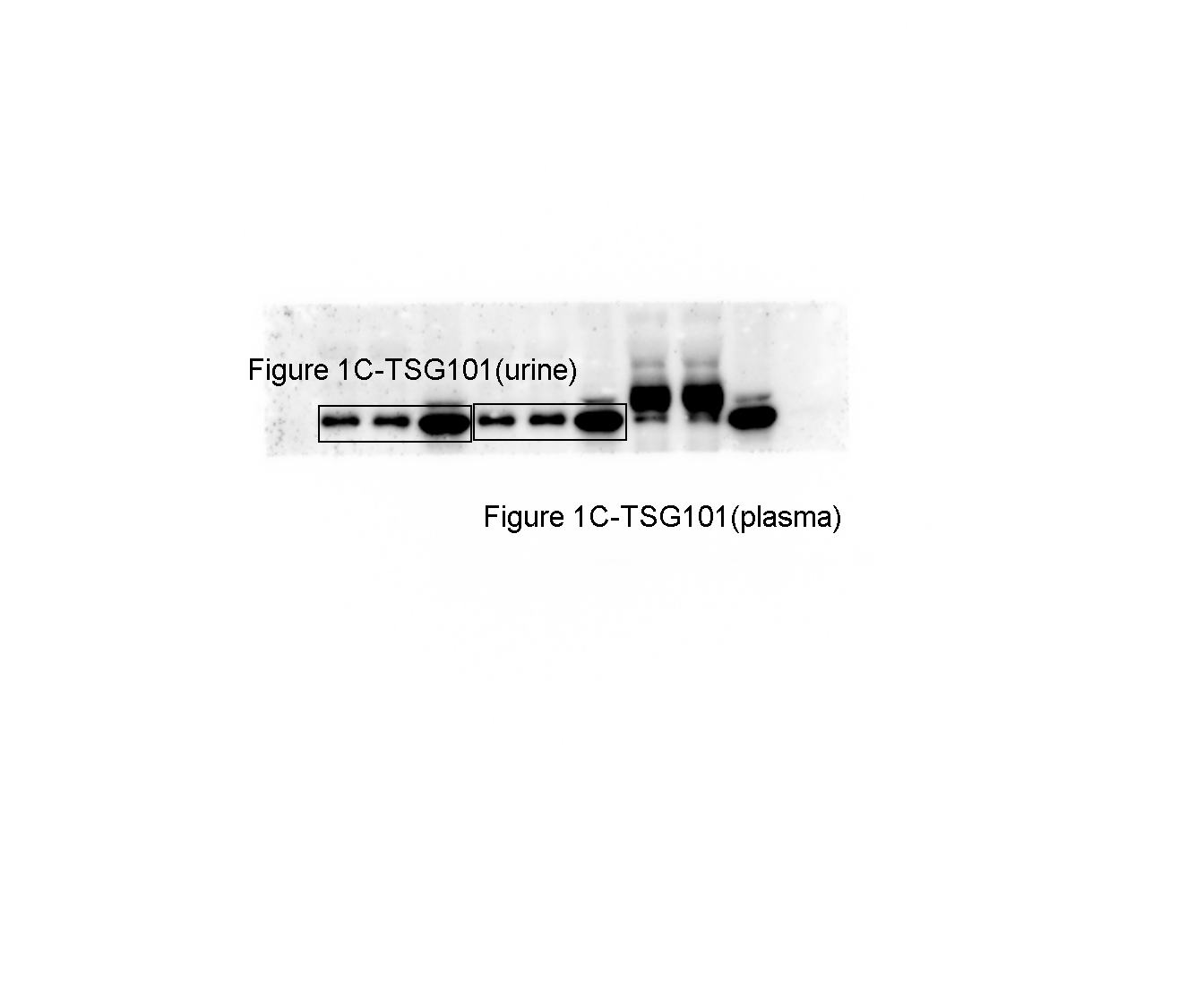

Supplement: Supplementary file 1 [file cancers-15-05305-s001.zip › cancers-15-05305-s001/File S1/Figure 1Cand 1F-TSG101.tif]

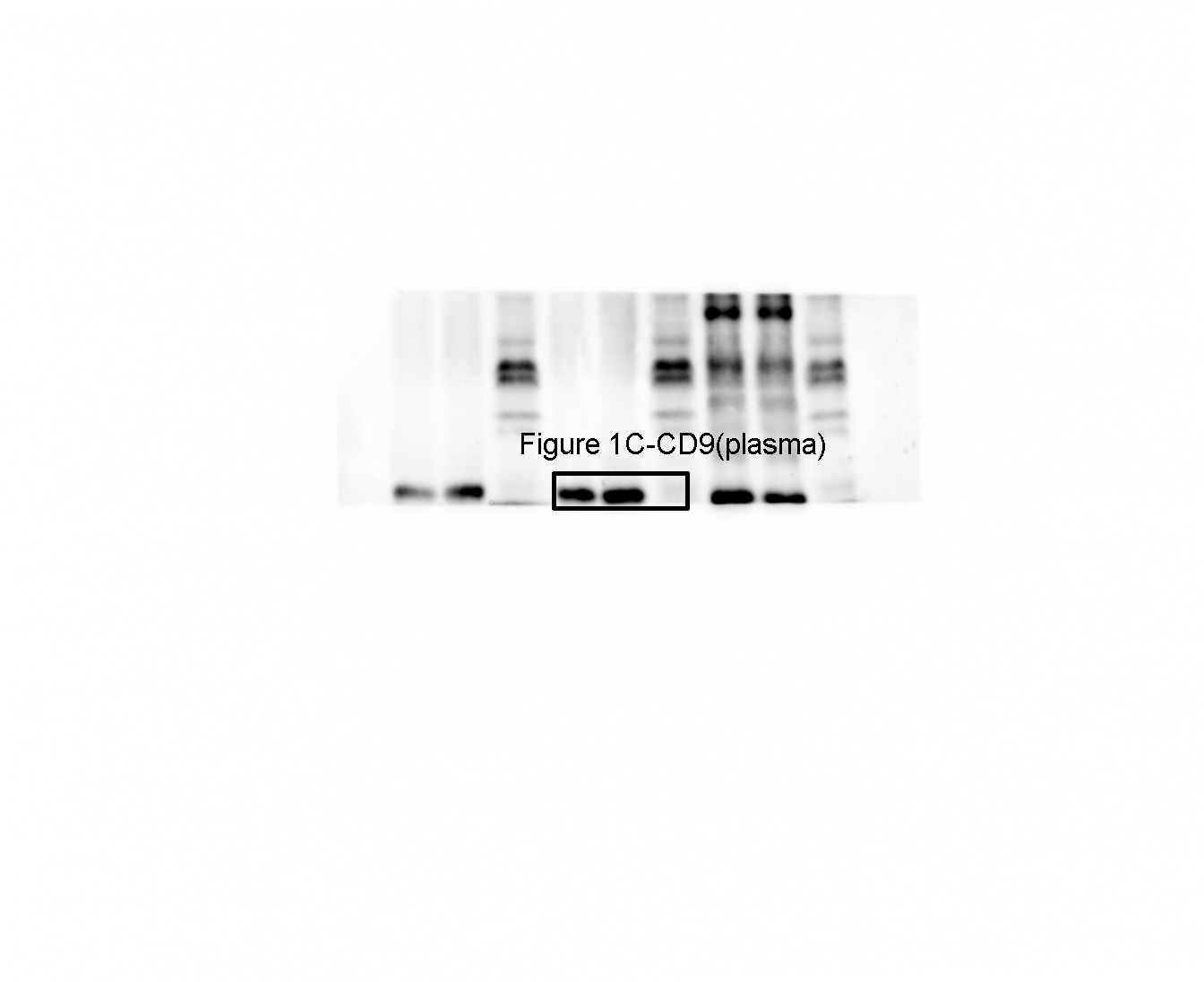

Supplement: Supplementary file 1 [file cancers-15-05305-s001.zip › cancers-15-05305-s001/File S1/Figure 1C-CD9(plasma).tif]

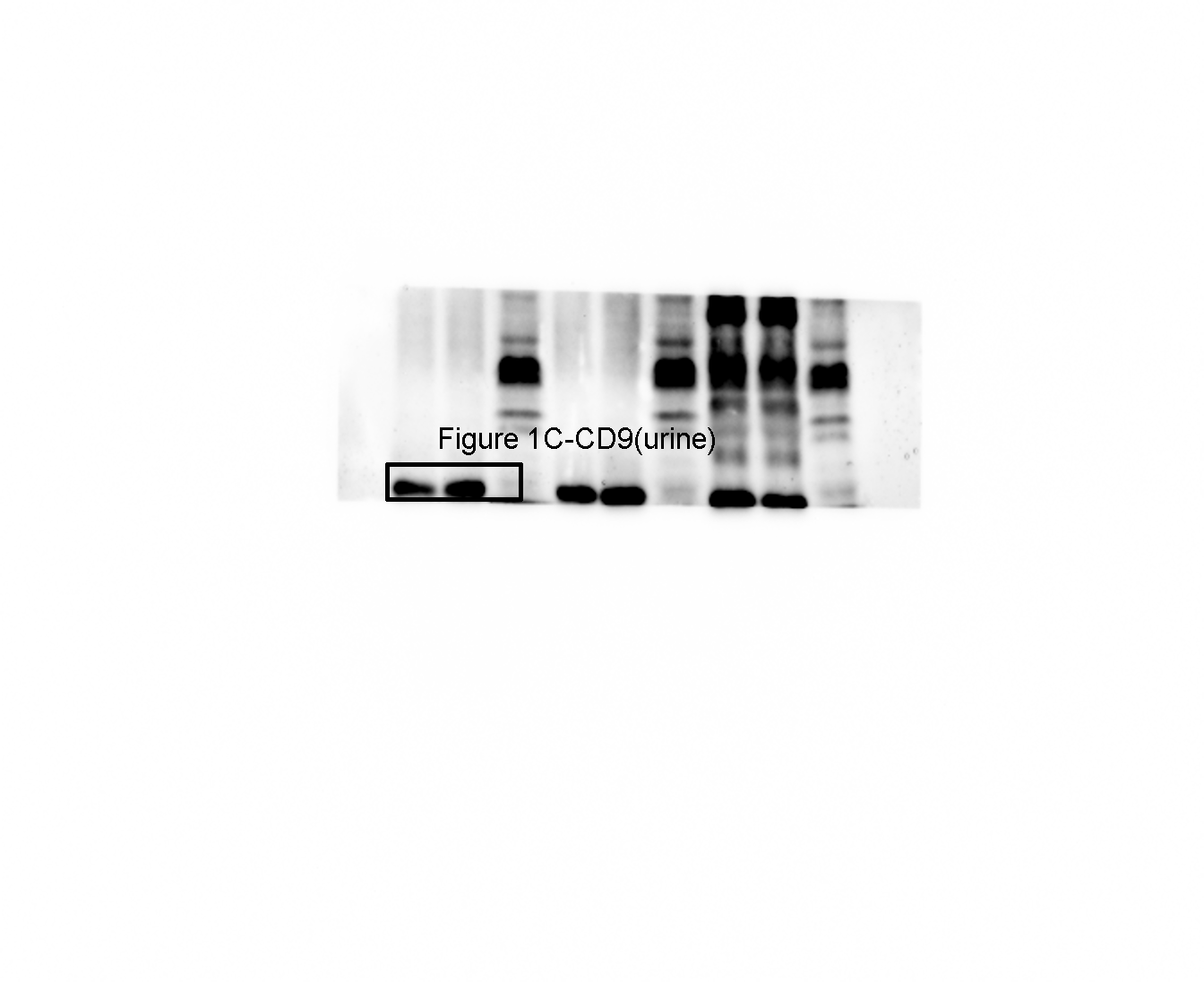

Supplement: Supplementary file 1 [file cancers-15-05305-s001.zip › cancers-15-05305-s001/File S1/Figure 1C-CD9(urine).tif]

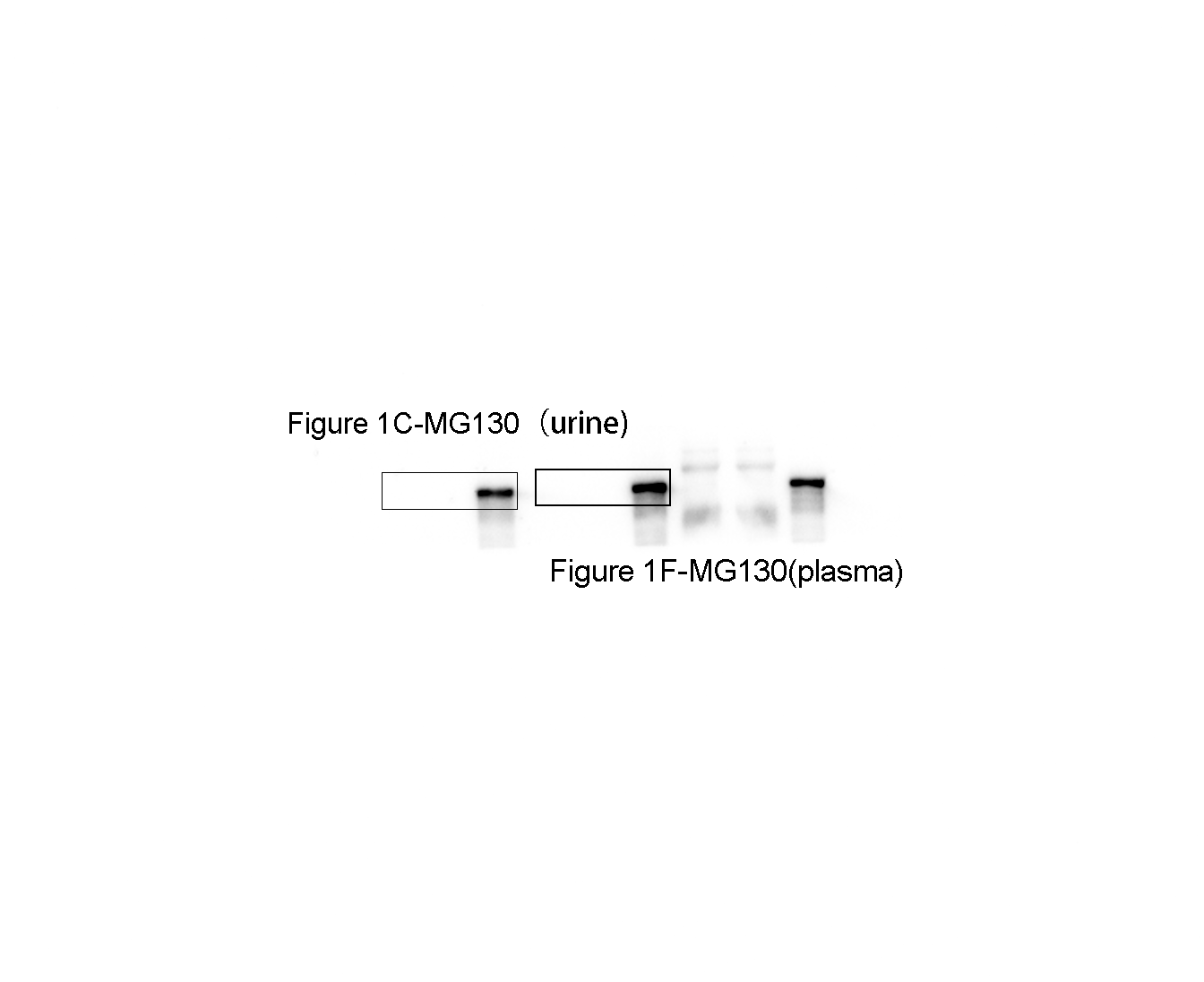

Supplement: Supplementary file 1 [file cancers-15-05305-s001.zip › cancers-15-05305-s001/File S1/Figure 1C-GM130.tif]

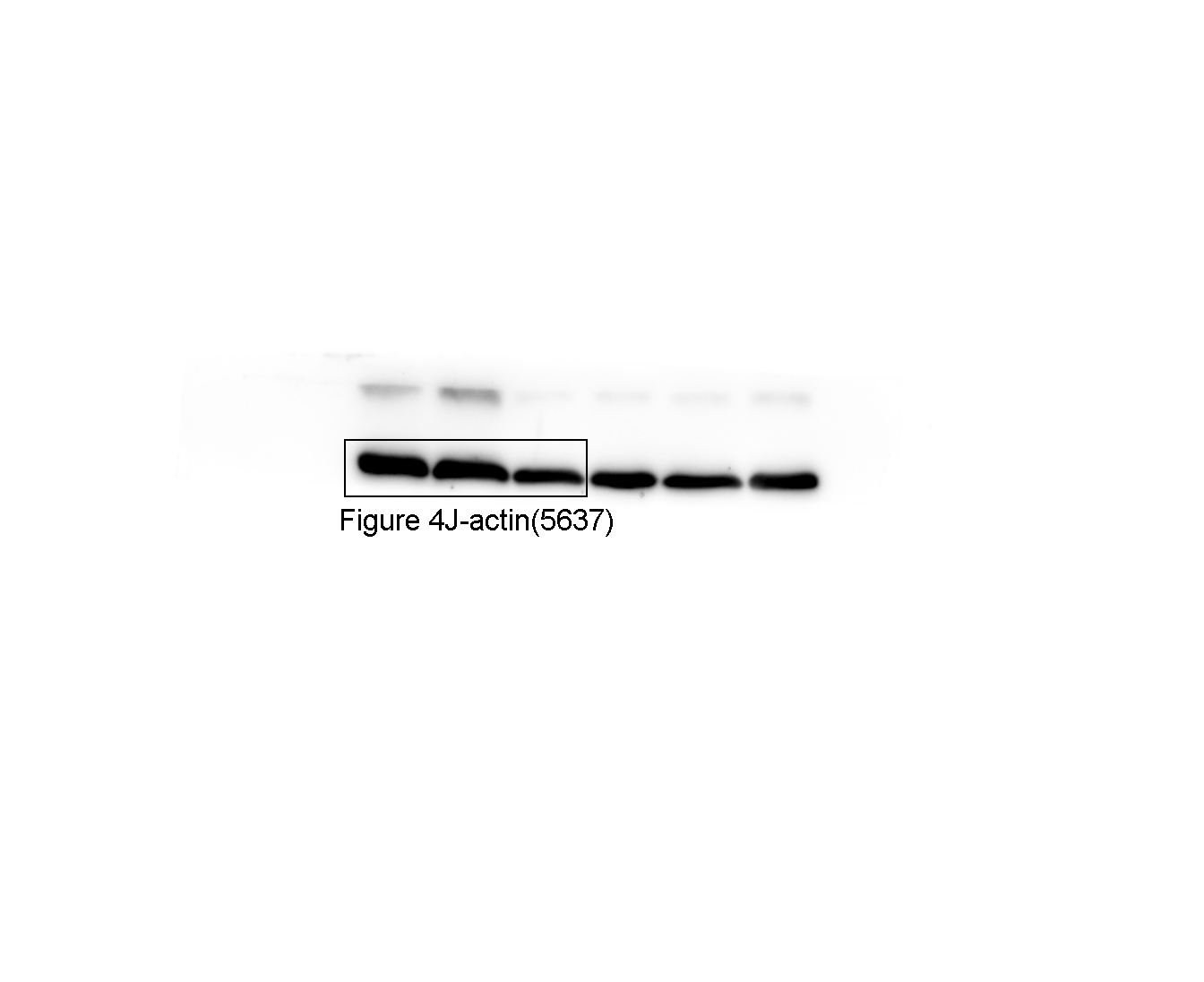

Supplement: Supplementary file 1 [file cancers-15-05305-s001.zip › cancers-15-05305-s001/File S1/Figure 4J-actin(5637).tif]

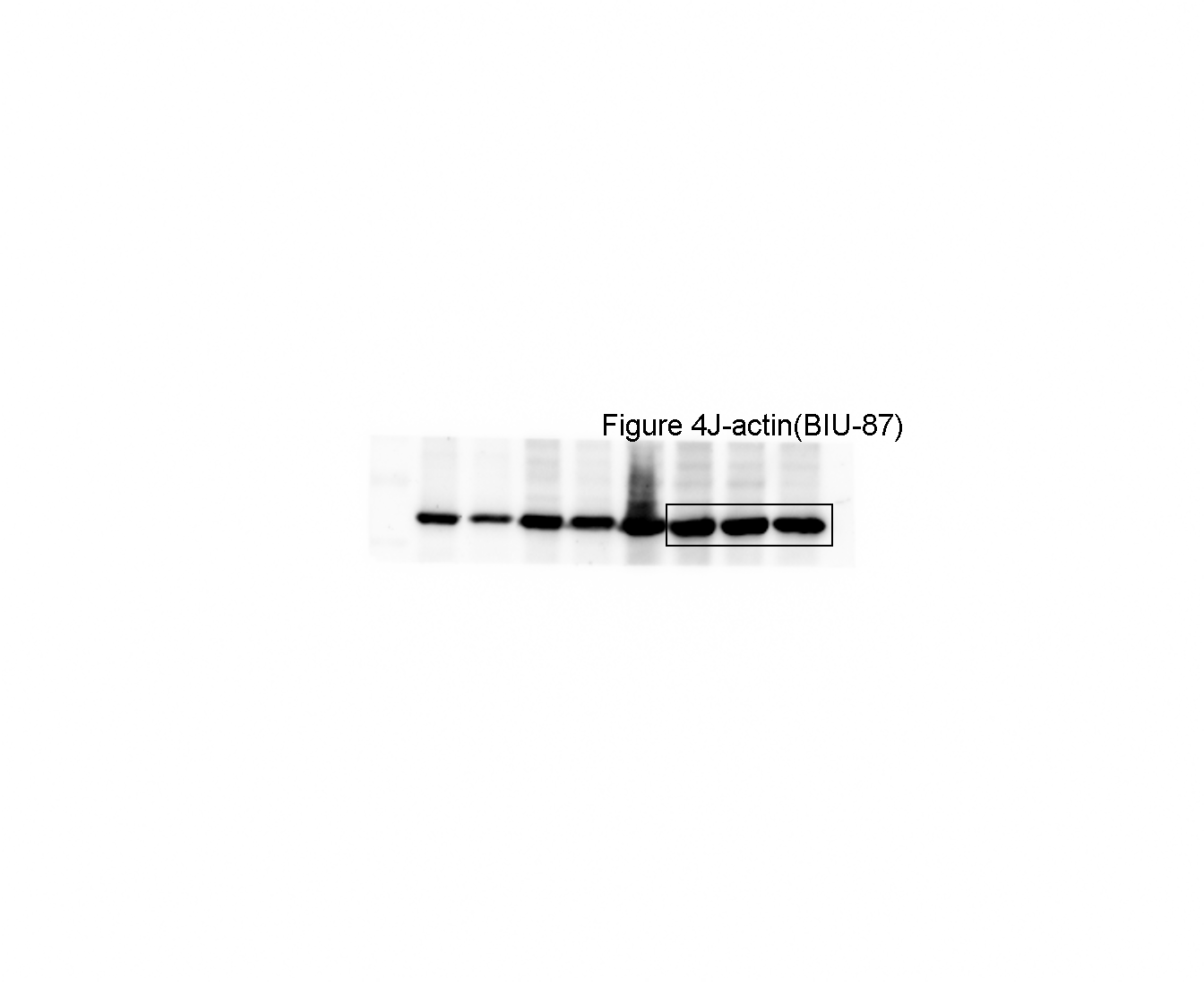

Supplement: Supplementary file 1 [file cancers-15-05305-s001.zip › cancers-15-05305-s001/File S1/Figure 4J-actin(BIU-87).tif]

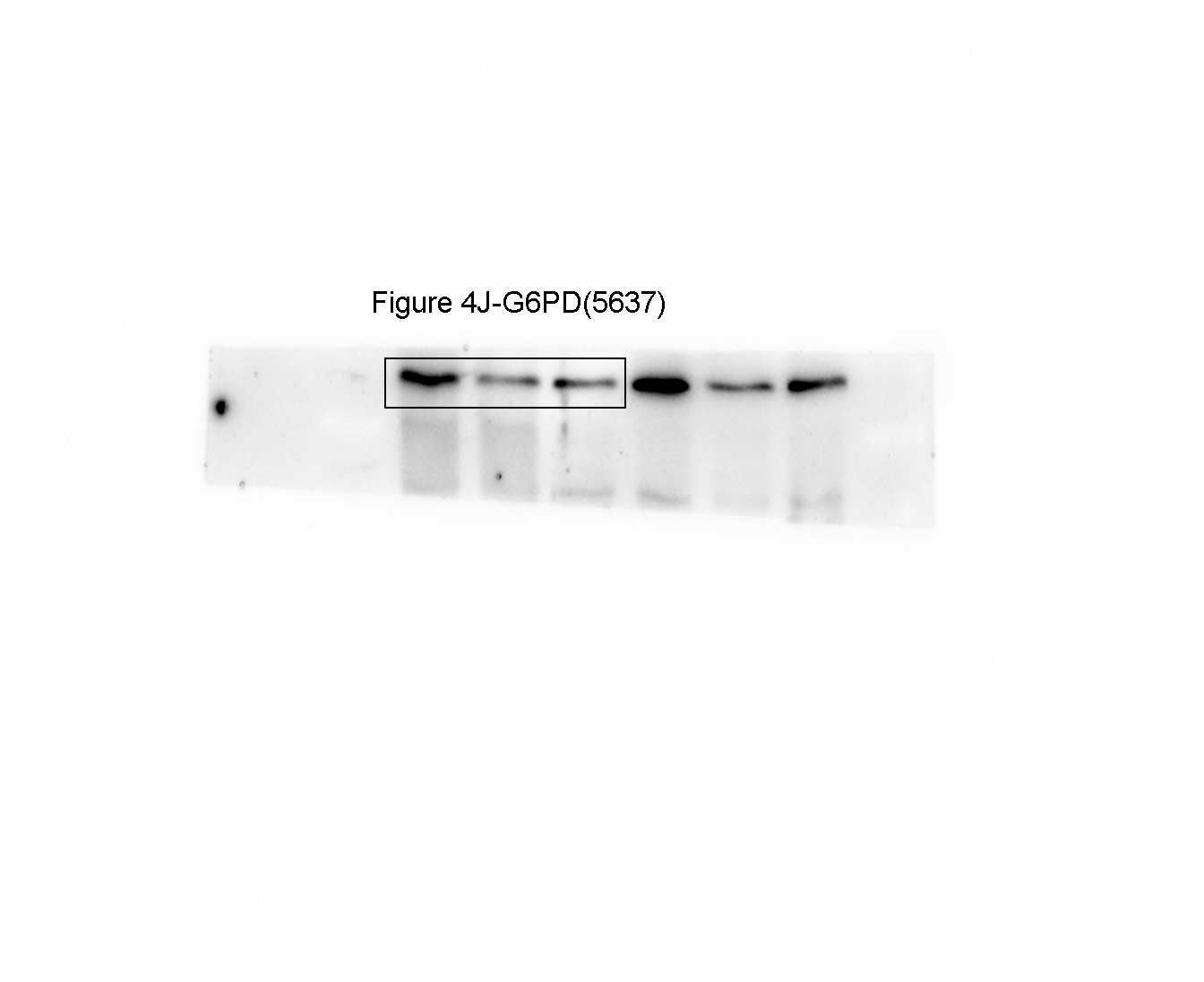

Supplement: Supplementary file 1 [file cancers-15-05305-s001.zip › cancers-15-05305-s001/File S1/Figure 4J-G6PD(5637).tif]

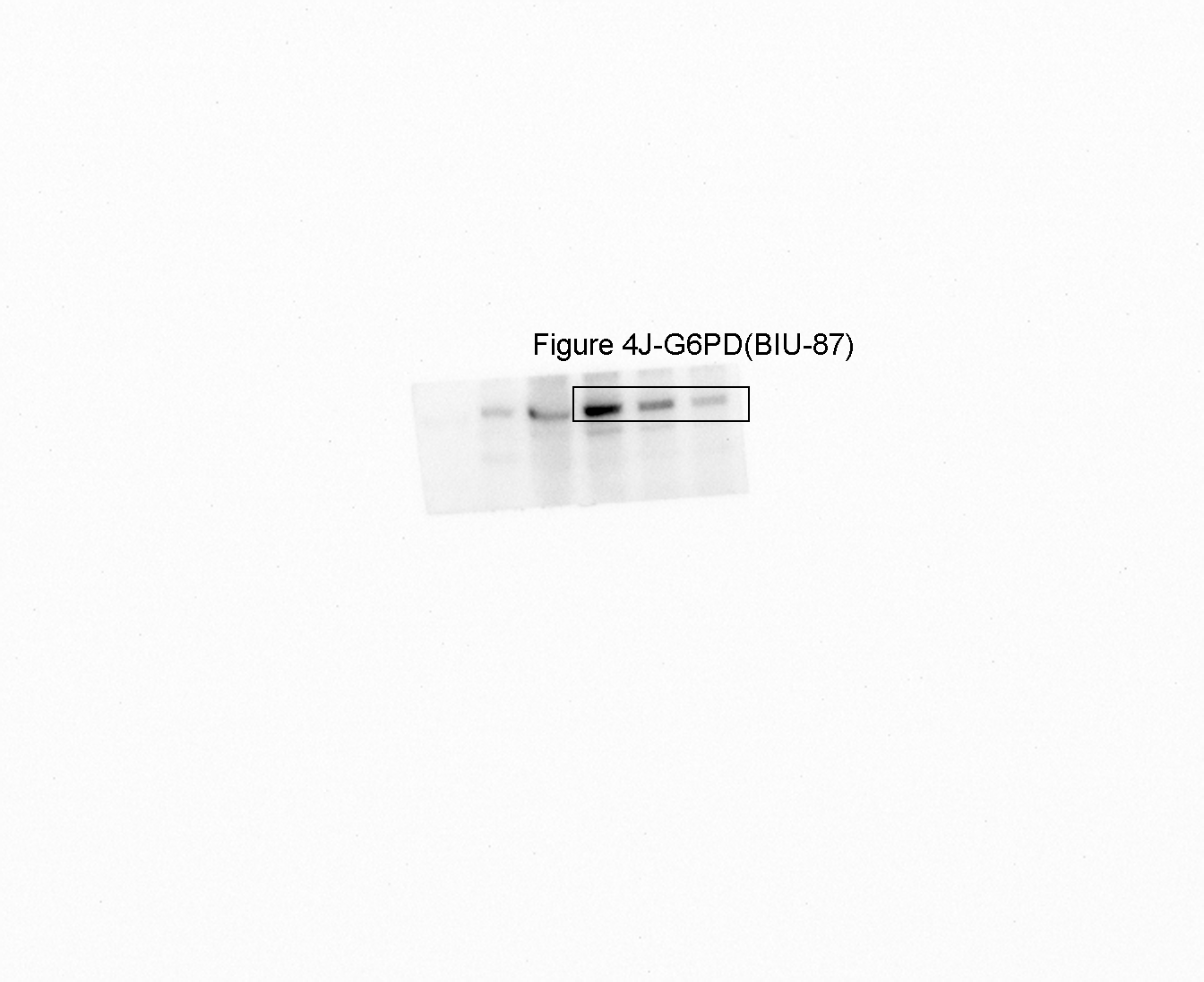

Supplement: Supplementary file 1 [file cancers-15-05305-s001.zip › cancers-15-05305-s001/File S1/Figure 4J-G6PD(BIU-87).tif]

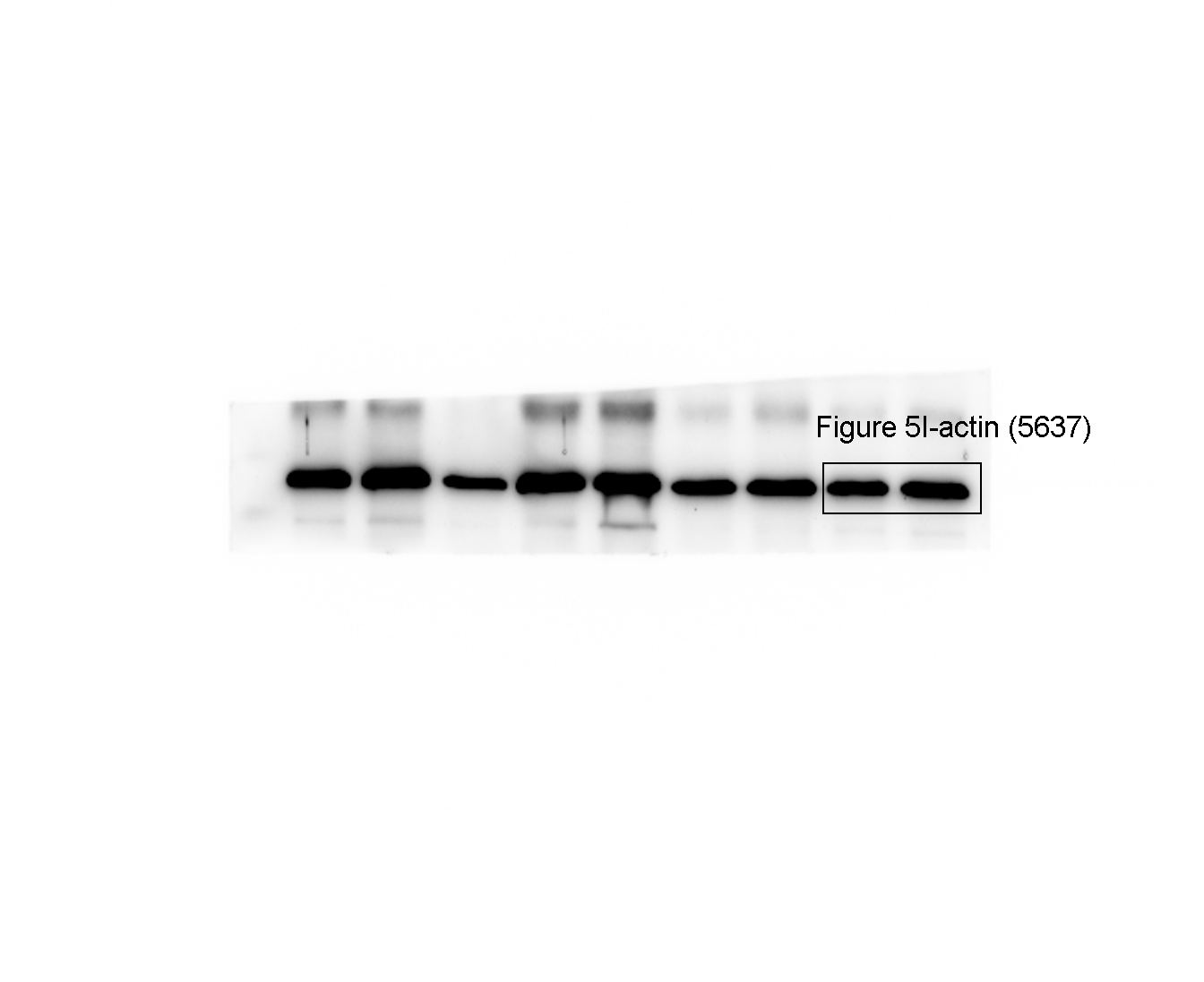

Supplement: Supplementary file 1 [file cancers-15-05305-s001.zip › cancers-15-05305-s001/File S1/Figure 5I-actin (5637).tif]

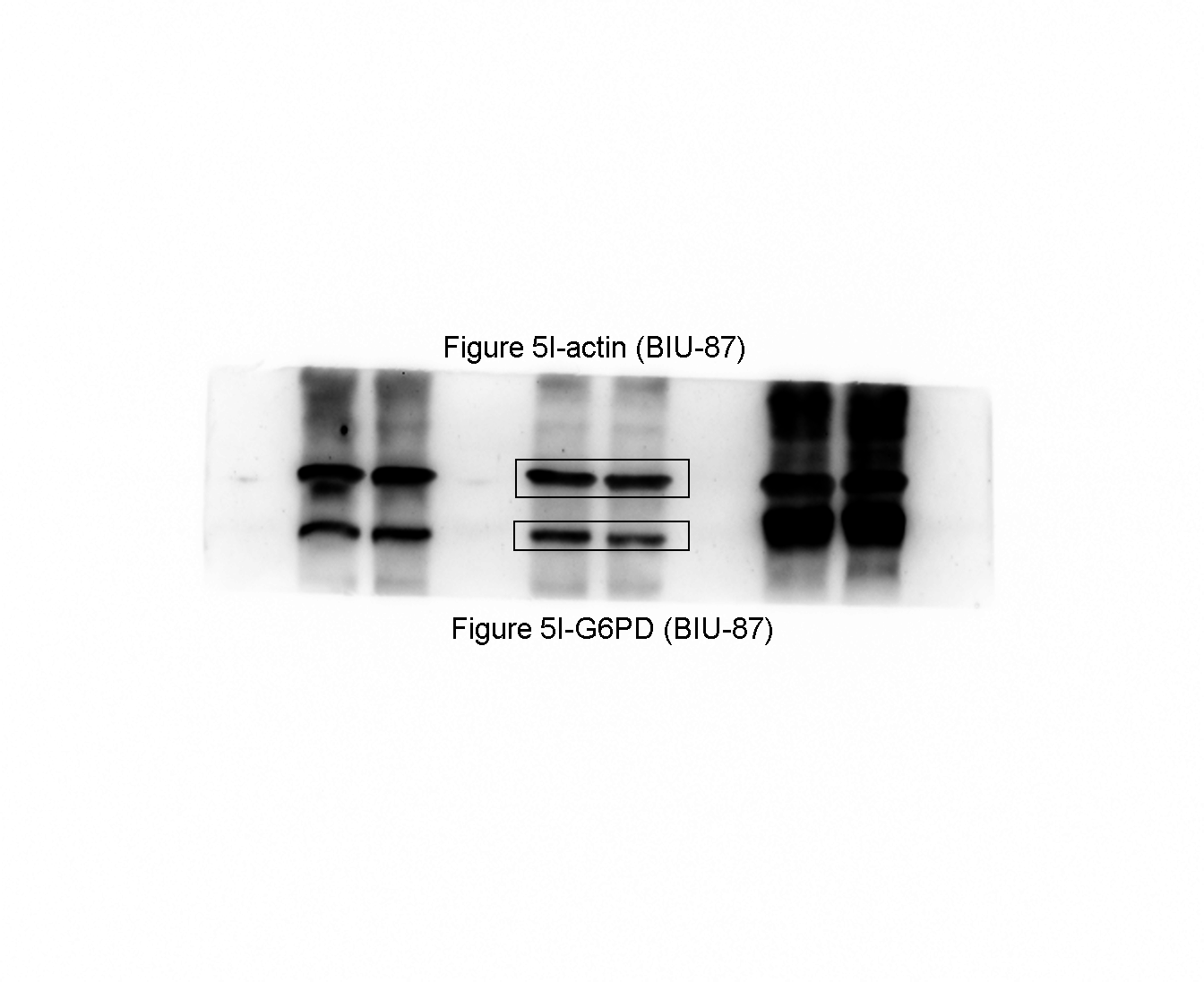

Supplement: Supplementary file 1 [file cancers-15-05305-s001.zip › cancers-15-05305-s001/File S1/Figure 5I-actin-G6PD (BIU-87).tif]

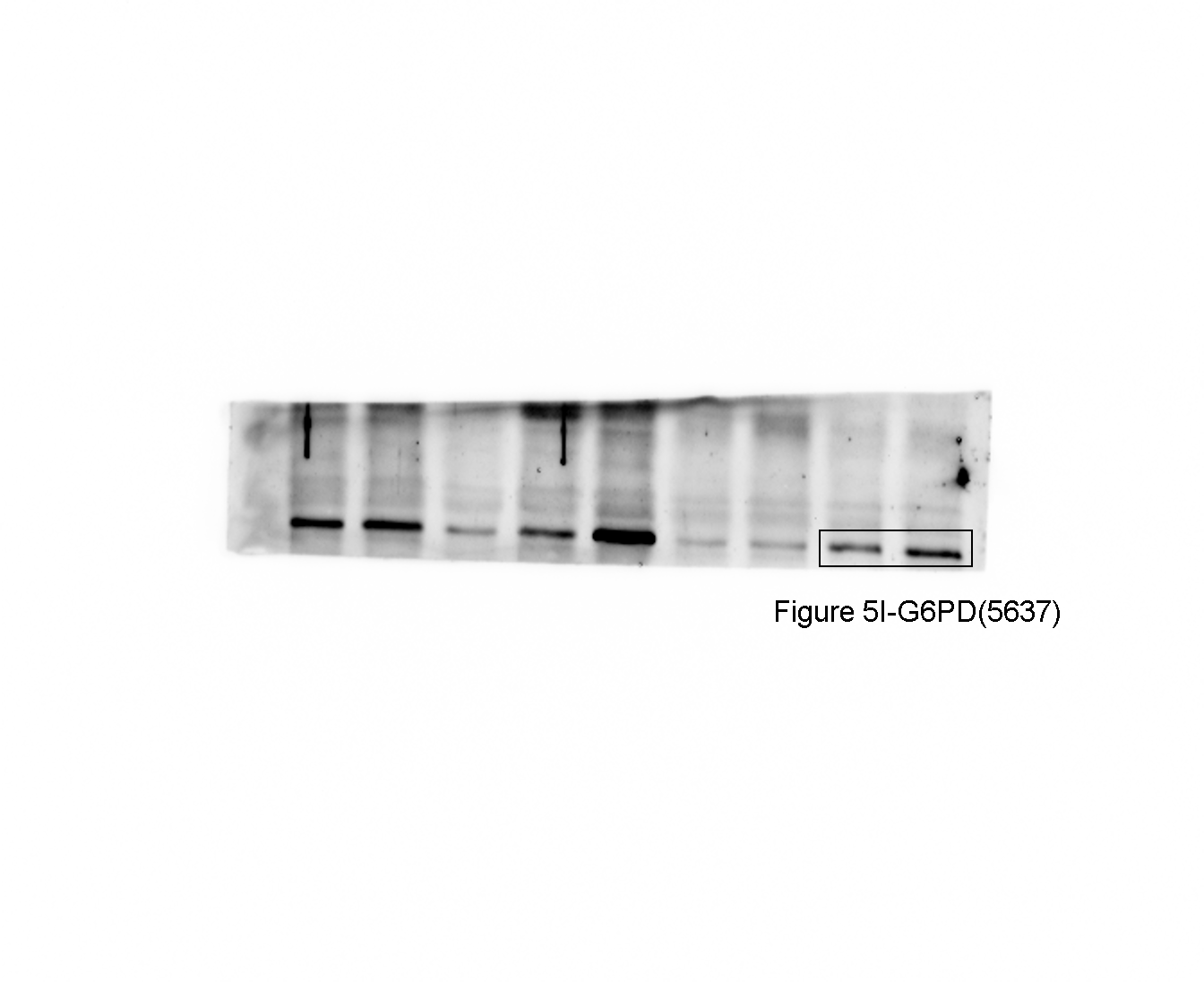

Supplement: Supplementary file 1 [file cancers-15-05305-s001.zip › cancers-15-05305-s001/File S1/Figure 5I-G6PD(5637).tif]

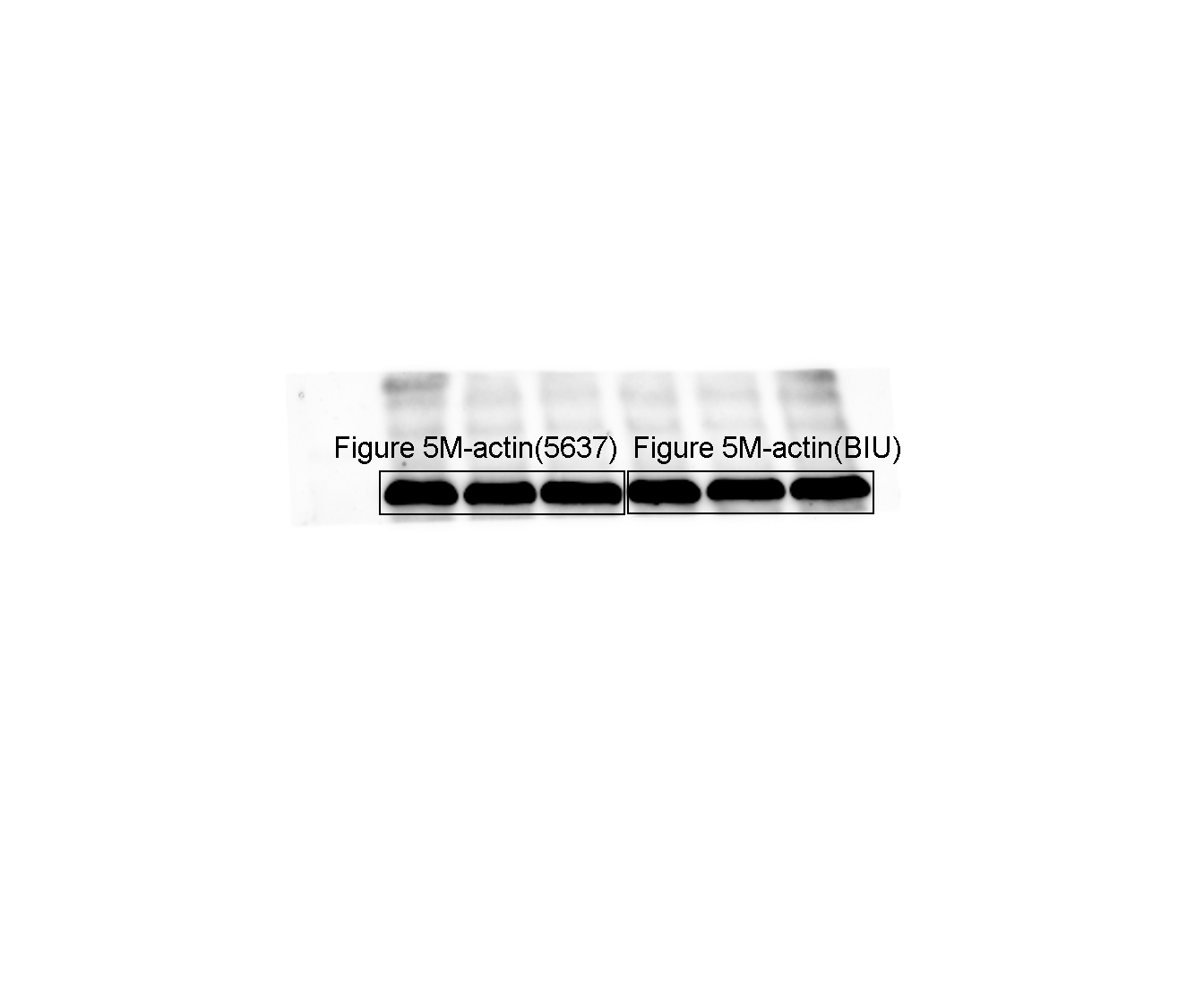

Supplement: Supplementary file 1 [file cancers-15-05305-s001.zip › cancers-15-05305-s001/File S1/Figure 5M-actin.tif]

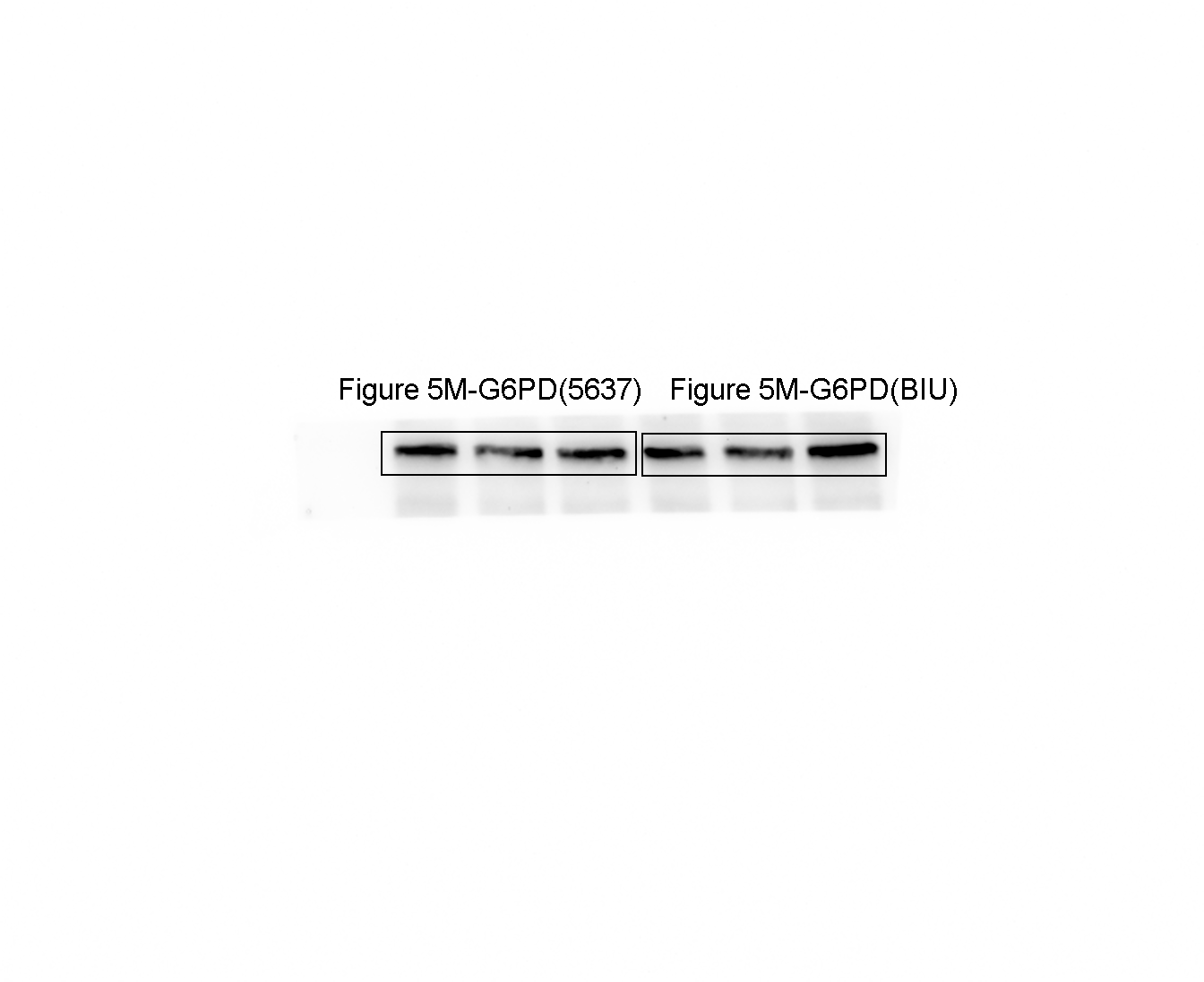

Supplement: Supplementary file 1 [file cancers-15-05305-s001.zip › cancers-15-05305-s001/File S1/Figure 5M-G6PD.tif]
